# Dynamic Pressure Measurements During Vitrectomy in a Model of the Eye

**DOI:** 10.1167/tvst.11.5.21

**Published:** 2022-05-18

**Authors:** Irene Nepita, Alessandro Stocchino, Andrea Dodero, Maila Castellano, Mariantonia Ferrara, Mario R. Romano, Rodolfo Repetto

**Affiliations:** 1 Nanoscopy and NIC@IIT, Istituto Italiano di Tecnologia, Genoa, Italy; 2 Department of Civil and Environmental Engineering, Hong Kong Polytechnic University, Hong Kong; 3 Adolphe Merkle Institute, University of Fribourg, Fribourg, Switzerland; 4 Department of Chemistry and Industrial Chemistry, University of Genoa, Genoa, Italy; 5 Newcastle Eye Centre, Royal Victoria Infirmary, Newcastle upon Tyne, UK; 6 Department of Biomedical Sciences, Humanitas University, Pieve Emanuele–Milan, Italy; 7 Ophthalmology Department, Humanitas Gavazzeni-Castelli, Bergamo, Italy; 8 Department of Civil, Chemical and Environmental Engineering, University of Genoa, Genoa, Italy

**Keywords:** IOP measurements, vitrectomy, pressure compensation, artificial vitreous

## Abstract

**Purpose:**

To accurately evaluate pressure changes during vitrectomy in a rigid model of the vitreous chamber and to test the efficiency of the EVA phacovitrectomy system (Dutch Ophthalmic Research Center) in terms of compensation of intraocular pressure variations.

**Methods:**

We tested 23-, 25-, and 27-gauge double-blade vitreous cutters in both vented global pressure control and automatic infusion compensation (AIC) modes in a vitreous chamber model, mimicking the real surgical procedure. Balanced salt solution and artificial vitreous, similar to the real vitreous body, were used. We tested both standard-flow (SF) and high-flow (HF) infusion systems, varying the infusion pressure between 20 and 40 mm Hg. In each experiment, flow rate was also measured.

**Results:**

Pressure drop was rapidly and efficiently compensated when 23- and 25-gauge cutters were used in AIC mode, with infusion pressures ranging between 30 and 55 mm Hg. The 27-gauge cutter was less efficient in compensating pressure variations. Pressure fluctuations related to the high-frequency motion of the cutter blade were small compared to the overall pressure variations. The use of the HF infusion system resulted in larger flow rates and lower pressure changes compared to the SF infusion system.

**Conclusions:**

Despite the rigid material of the model, the present pressure measurements are in line with previous studies performed on porcine eye. The use of AIC mode compensates intraoperative pressure drops efficiently, with both 23- and 25-gauge cutters. The HF infusion system is more efficient than the SF infusion system.

**Translational Relevance:**

The AIC infusion mode efficiently compensates intraoperative pressure drops, in both 23- and 25-gauge experimental vitrectomy. The HF infusion system resulted in larger flow rate and lower pressure changes.

## Introduction

Understanding the fluidics of pars plana vitrectomy is crucial to optimize surgery, in terms of both effectiveness and safety.^1^ From the practical point of view, optimization of fluidics can be achieved by maximizing the flow rate and minimizing pressure fluctuations.^2^ A constant intraocular pressure (IOP) is essential to ensure intraoperative stability of the vitreous chamber. Indeed, large IOP fluctuations may lead to serious intraoperative and postoperative complications, such as choroidal detachment, vitreous hemorrhage, expulsive choroidal hemorrhage, and retinal and optic nerve ischemia.^3^^–^^9^ It is also known that an intermittent flow generates fluid accelerations, with consequent rapid pressure variations within the vitreous chamber, which may result in pulsatile tractions on the retina and iatrogenic retinal damage.^10^^–^^13^ Moreover, the mean ocular perfusion pressure (MOPP) strictly depends on the intraoperative IOP, and a sustained IOP increase during vitrectomy can lead to a significant decrease of the MOPP and harmful effects due to the ocular hypoperfusion.^14^

Several studies have evaluated IOP fluctuations during vitrectomy, in both animal and human eyes, and values ranging from 0 to 120 mm Hg have been reported.^15^^–^^19^ In light of these findings, several modern vitrectomy devices are equipped with pressure compensation systems, aimed at actively counteracting IOP changes during surgery. Their efficacy has been studied in experimental vitrectomy by various groups, in most cases using Alcon vitrectomy systems (Alcon Ltd, Fort Worth, TX, USA).^15^^,^^16^^,^^20^^–^^24^

The purpose of the present study was to investigate and quantify dynamic intraocular pressure variations in a vitreous chamber model and to assess the efficiency in maintaining baseline pressure values during vitrectomy, using the EVA phacovitrectomy system provided by the Dutch Ophthalmic Research Center (DORC), Zuidland, The Netherlands. No previous similar tests are reported for this system. In addition, we performed flow rate experiments, with the aim of assessing the performance of the EVA high-flow (HF) infusion connection compared with the standard-flow (SF) one. In the traditional SF configuration, the end of the infusion line is inserted into the trocar-cannula, whereas in the HF configuration, the infusion tube is connected to the external part of the trocar-cannula, resulting in an increased inner diameter and, consequently, reduced hydraulic resistance.

We adopted a model of the vitreous chamber with a realistic geometry and performed experiments both with balanced salt solution (BSS) and with a substance obtained as a solution of agar–agar polysaccharide in pure water (artificial vitreous [AV]), which has viscoelastic properties similar to those of the human vitreous.

## Materials and Methods

### Experimental Setup and Measuring Techniques

**Figure 1.**
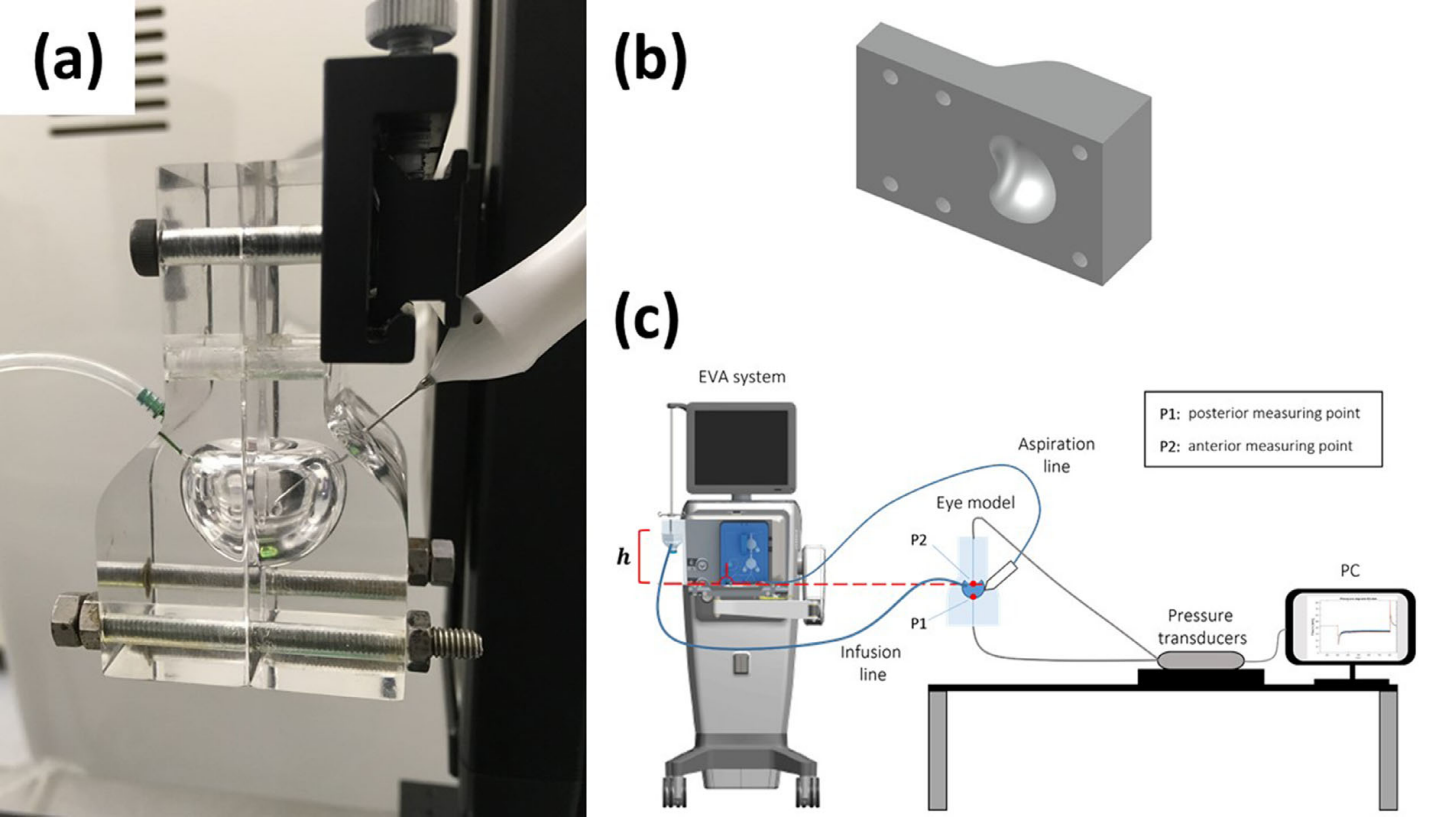
(a) Picture of the assembled eye model connected with infusion and aspiration lines; (b) cross section of the physical model; (c) sketch of the experimental apparatus.

Experiments were carried out in a transparent physical model of the vitreous chamber at real scale and with a realistic geometry.^25^^–^^29^ The model is made of rigid Plexiglas and has been produced with a three-dimensional CNC milling machine. It consists of two identical parts, each representing half of the vitreous chamber (see Fig. 1a). A three-dimensional representation of half of the model showing the shape of the vitreous chamber is reported in Figure 1b. The model was attached to a rigid support, held in a “face-up” position, and connected to infusion and aspirations lines, thus mimicking the real surgical procedure. Infusion and aspiration lines were located approximately in correspondence of the “pars plana,” as shown in Figure 1. Two pressure transducers (Viatran model 422, Viatran corporate, Tonawanda, NY, USA) were connected to the eye model, which can register pressures in a range of 0 to 155 mm Hg, with a nonlinearity less than ±0.06% of the full range and a repeatability accuracy of less ±0.06% of the full range The pressure measuring points were located along the axis of symmetry of the vitreous chamber, one at the center of the posterior surface of the lens (P2) and one at the posterior pole of the chamber model (P1), as shown in Figure 1c. The connections between the eye model and the pressure transducers were made through identical silicon tubes and plastic connectors. Pressure signals have been acquired using a digital acquisition system (National Instruments, Austin, Texas, USA) controlled by a LabVIEW graphical user interface. The acquisition frequency was set to 1.5 KHz for all experiments, which is more than 10 times higher than the maximum cutting rate frequency used during experiments.

Once the vitreous chamber model was filled with the working fluid, AV, or BSS, a typical experiment consisted of three phases:

1. Phase 1: in the first phase, continuous infusion was switched on, and there was no aspiration by the vitreous cutter; thus, the eye model was pressurized but there was no fluid motion.
2. Phase 2: in the second phase, aspiration was activated, and in the absence of compensation, this caused a sudden pressure drop in the eye model. During this phase, infusion and aspiration were both active.
3. Phase 3: in this last phase, aspiration was stopped, whereas the infusion line was still active; consequently, the pressure returned to the initial value of phase 1 and fluid flow stopped.

In our experimental setup, in static conditions, the two pressure transducers registered slightly different pressure values (*≈*1.1 mm Hg), owing to the elevation difference between the two measuring points in the eye model (see Fig. 1c for the relative position of the two transducers). The EVA system controls the infusion pressure by imposing a prescribed pressure value, *p*_0_, in the air of the infusion bottle. Thus, the pressure in the vitreous chamber in static conditions is equal to *p*_0_ + γ*h*, where γ is the specific weight of the fluid and *h* is the height between the free surface in the bottle and the level of the vitreous chamber (see Fig. 1c). Since the level of the free surface in the bottle varied from one experiment to the other (*h* approximately ranged from 10 to 19 cm), with the aim of comparing the measurements performed by varying the controlling parameters, the corresponding hydrostatic pressure contribution has been subtracted from the measured pressures.

Pressure data have been postprocessed as follows: first, the starting and ending times of the various phases of the experiments were identified; then, pressure averaged values (*P_b_* and *P_a_*, corresponding to the phases 1 and 2, respectively) and corresponding standard deviations were computed. We also computed the average pressure jump, Δ*P*, defined as Δ*P* = *P_b_* − *P_a_*.

For each experiment, we measured the flow rate using a remotely controlled precision digital scale (A&D compact scale, EK-1200G, Higashi-ikebukuro, Toshima-ku, Tokyo, Japan). The aspirated liquid was collected in a beaker positioned on the scale, and its mass, *M*(*t*), was recorded in time, *t*, with a sampling frequency of 5 Hz. The time derivative of the signal, *dM*/*dt*, provides an estimate of the mass flow rate *Q_m_*, from which the volumetric flux *Q* can be obtained as *Q* = *Q_m_*/ρ, with ρ being fluid density. This calculation was done performing a linear regression of fluid mass in time *M*(*t*); the slope of the line represents *Q_m_*. The coefficient of determination *R*^2^ of the linear regression was found on average to be 0.9986 *±* 0.001, which means that the signals were linear to a very good degree of approximation. The acquisition rate of the digital scale was not high enough to accurately measure the temporal variation of the flow rate. Thus, we obtained only the time-averaged flow rate.

We tested 23-, 25-, and 27-gauge two-dimensional cutting (TDC) vitreous probes, in both vented global pressure control (VGPC) and automatic infusion compensation (AIC) modes of the EVA vitrectomy system. The EVA system has been operated in the “core vitrectomy” modality, imposing a vacuum aspiration pressure. In VGPC mode, a fixed infusion pressure is imposed (baseline pressure), whereas in AIC mode, minimum and maximum infusion pressures are set and the device switches from one value to the other, with the aim of compensating pressure fluctuations during surgical maneuvers. In all experiments performed in AIC mode, the maximum infusion pressure value was set to 55 mm Hg.

For the 23- and 25-gauge TDC cutters, we tested both the SF and HF infusion systems, whereas only the HF system was tested for the 27-gauge TDC cutter. The operating conditions imposed during all experiments are reported in Table 1. We performed a total number of 68 experiments, using both BSS and AV. Details of AV preparation and its mechanical characterization are provided in the following section. All experiments with AV have been performed with the HF infusion connection.

**Table 1.** Experimental Parameters

Infusion System	TDC Cutter	Aspiration Pressure (mm Hg)	Cut Rate (cpm)	Working Fluid	Infusion Pressure (mm Hg)	Infusion Type	No. of Experiments
VGPC	23	400	6000	BSS	20-25-30-35-40	SF/HF	10
VGPC	25	600	6000	BSS	20-25-30-35-40	SF/HF	10
VGPC	27	600	6000	BSS	20-25-30-35-40	HF	5
VGPC	23	600	6000	AV	20-30-40	HF	6
VGPC	25	600	6000	AV	20-30-40	HF	6
AIC	23	400	6000	BSS	20-25-30-35-40	SF/HF	10
AIC	25	600	6000	BSS	20-25-30-35-40	SF/HF	10
AIC	27	600	6000	BSS	20-25-30-35-40	HF	5
AIC	23	400	6000	AV	20-30-40	HF	3
AIC	25	600	6000	AV	20-30-40	HF	3

In AIC mode, the infusion pressure reported in the table refers to the minimum value. The maximum infusion pressure value was always set to 55 mm Hg.

Repeatability of the experiments has been tested repeating one test seven times, with a specific set of parameters. The relative error on the pressure in the *n*th test *P_n_* with respect to the average value, $\left(P_{n}-\bar{P}\right)/\bar{P}$, with $\bar{P}=\sum_{n=1}^{N}\bar{P}/N$, was found to be always less than *±*4%. Moreover, the standard deviation of the single measurement is *≈*2*.*5% of the average value, indicating a fairly good repeatability of measurements.

Finally, the duration of a single experiment varied between 210 and 250 seconds, depending on the working fluid, cutter size, and infusion mode.

### Artificial Vitreous Preparation and Rheological Tests

Sigma-Aldrich (St. Louis, MO, USA) agar–agar polysaccharide was used to prepare AV fluids. Agar–agar is a natural polysaccharide, widely used for biomedical applications.^30^^,^^31^ Polymer powder was first added, at a concentration of 0.1%, 0.15%, or 0.2% (w/w), to deionized water and then completely solubilized by heating the samples with a microwave. The solutions were heated to the boiling point for a few seconds, vigorously mixed, and brought to boil again. The samples were finally allowed to slowly cool down at room temperature and then preserved at 4°C to avoid polymer degradation.

The viscoelastic behavior of the prepared AV was assessed via a rotational rheometer MCR 301 (Anton Paar GmbH, Graz, Austria) equipped with a Peltier heating system. A parallel-plate geometry with a diameter of 50 mm (PP-50) was used, setting the gap to 0.5 mm. The temperature was set to 20.0 ± 0.2°C (the temperature at which experiments have been performed) and to 37.0 ± 0.2°C. A normal force of 0 N was applied to the samples in order to ensure the measurement of the linear properties of agar gels.^32^ In previous works,^13^^,^^33^ steady-state viscosity measurements were carried out to explore the rheological behavior of artificial fluids with different properties. Here, owing to the gel-like nature of the prepared agar-based AV, we instead performed only oscillatory tests. Amplitude sweep tests were first performed to determine the linear viscoelastic region of the materials, and they were carried out in the strain range of 0.01% to 10%, at frequency of ω = 1 Hz. Frequency sweep tests were then used to evaluate the viscoelastic response of the samples at a strain of 1% (within the linear region) and varying the frequency between 0.1 and 10 Hz. Oscillatory tests provide the values of the storage (*G*′) and loss (*G*′′) moduli, as well as the complex viscosity modulus, |η| = (*G*^′2^ + *G*^′′2^)^1/2^/ω. *G*′ is a measure of the elasticity of the material, *G*′′ quantifies its capability to dissipate energy, and η is directly related to its tendency to flow.

## Results

### Rheological Measurements

For all polymer concentrations, AVs have a gel-like behavior in the whole range of investigated frequencies, with a larger value of the elastic modulus compared to the loss one. Increasing the agar–agar concentration leads to an increase of both viscoelastic moduli, as well as of the complex viscosity of the prepared AVs. This is because a higher number of polymer chains leads to a “denser” polymer network, characterized by closer crosslinking points and entanglements.^34^^,^^35^ Moreover, regarding the complex viscosity, all samples show a decrease of η as the frequency is increased, which is consistent with their gel-like behavior. Such a finding is related to the fact that, as the frequency increases, gels are subject to a progressively greater stress, able to break their microstructure (the physical crosslinking points between polymer chains), thereby facilitating their flowing.

**Figure 2.**
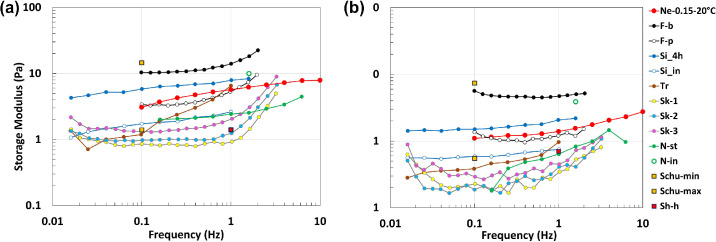
Storage G′ (a) and loss G′′ (b) moduli as a function of the testing frequency taken from the experiments on vitreous of various species. F-b, Filas et al.^37^ bovine vitreous; F-p, Filas et al.^37^ porcine vitreous; Ne-0.15-20°C, results of the present analysis; N-in, Nickerson et al.^38^ initial values, porcine vitreous; N-st, Nickerson et al.^38^ steady-state values, porcine vitreous; Schu-min and Schu-max, Schulz et al.^39^ minimum and maximum values; Sh-h, Shafaie et al.^4^^0^ human vitreous; S-i, da Silva et al.^41^ initial values, rabbit vitreous; Si-4h, da Silva et al.^41^ 4 hours after dissection, rabbit vitreous; Sk-i, Sharif-Kashani et al.^4^^2^ (i = 1; 2; 3 denotes different eyes), porcine vitreous; Tr, Tram et al.^43^ human vitreous.

The sample with a polymer concentration of 0.15% was selected for the experiments, as it was found to have viscoelastic properties similar to those of the real vitreous. Repetto and Dvoriashyna^36^ reported a fairly detailed picture of the existing measurements of real vitreous properties (animal and human). All these findings are shown in Figure 2, in terms of *G*′(ω) in Figure 2a and *G*′′(ω) in Figure 2b. In the figure, we also report our measurements for the solution selected for the experiments, which falls within the appropriate range for both *G*′(ω) and *G*′′(ω) (red curves).

### Pressure Measurements

**Figure 3.**
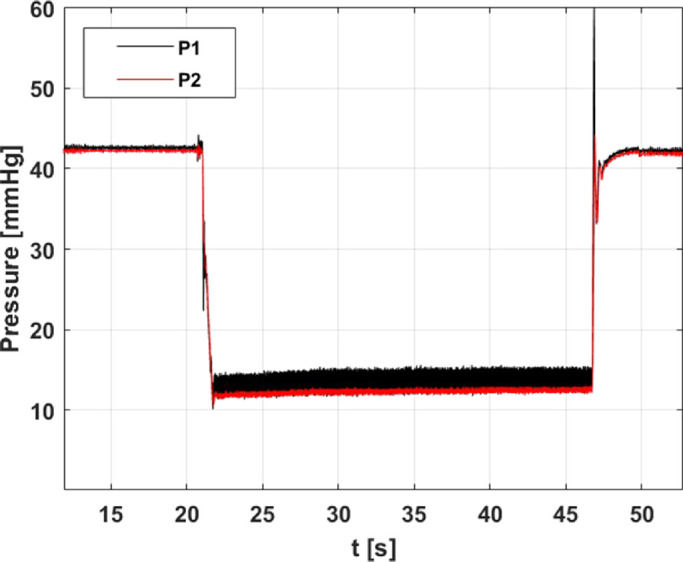
Example of pressure versus time signals, measured at two different positions of the eye model, using BSS in VGPC mode. *Black curve*: posterior position (P1); *red curve*: anterior position (P2).

During the phase 1 of each experiment, the system attained a constant pressure value that is considered the baseline for the experiment. When the pedal of the vitreous cutter was pressed, aspiration started (phase 2), and in VGPC mode, in the absence of pressure compensation, the pressure in the eye model dropped, owing to pressure losses along the system. Once the pedal was released, aspiration stopped (phase 3), and the pressure returned to the baseline value. Typically, in experiments with BSS, the pressure remained fairly constant during the aspiration phase. The amplitude of the high-frequency pressure fluctuations related to the cutter motion was larger at the posterior position of the vitreous chamber model (measuring point P1) than at the anterior one (measuring point P2). This is probably due to the generation of a jet that impinges the chamber wall opposite to the sensor located in front of the infusion line. Figure 3 reports an example of pressure measurements during a typical experiment with BSS; the three phases of the experiment are clearly recognizable.

**Figure 4.**
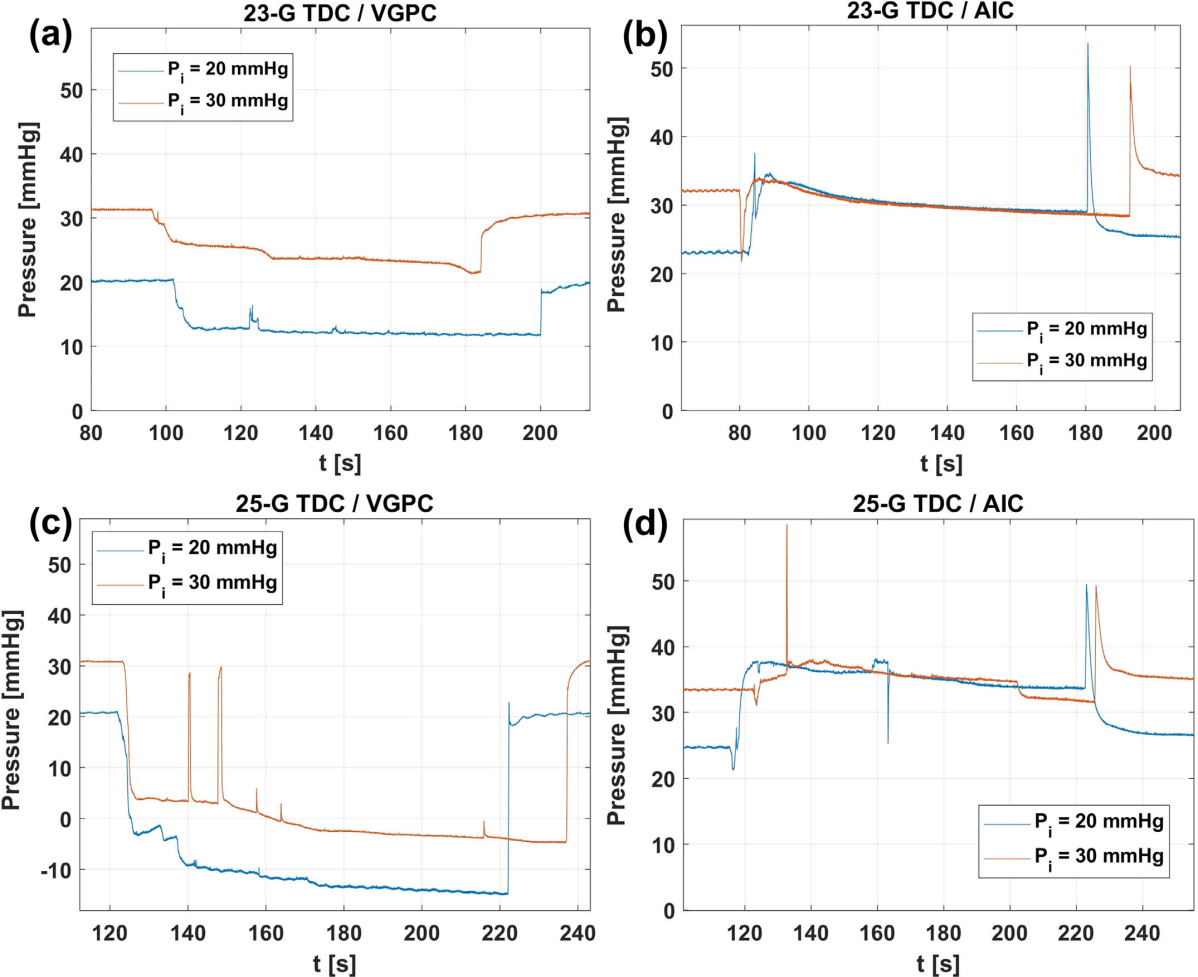
Pressure versus time as measured in experiments with AV: 23-gauge TDC cutter (a, b), 25-gauge TDC cutter (c, d); experiments in VGPC mode (a, c); experiments in AIC mode (b, d). In all panels, the *blue curves* correspond to infusion pressure *P_i_* = 20 mm Hg and the *orange curves* to 30 mm Hg. Signals acquired at position P2.

When we performed measurements with AV, the substance in the vitreous chamber model progressively changed properties during the experiment, since AV mixed up with the infused BSS, as it happens during the real surgery. This has consequences on the pressure attained in the eye model that are shown in Figures 4a and 4c, where we report results obtained with the 23-gauge TDC and 25-gauge TDC cutters in terms of pressure versus time. The pressure signals refer to the transducer connected to the anterior position of the vitreous chamber model (P2). In each panel, the two curves are relative to two different values of the infusion pressure. The figures show that, during aspiration (phase 1), in most experiments, the pressure progressively decreased in time. This is a consequence of the fact that viscosity of BSS is smaller than that of AV, and the more BSS enters the hydraulic circuit, the smaller the pressure is expected to be in the eye model, owing to an overall decrease of hydraulic head losses.

In all experiments, we observed that stopping the aspiration caused a pressure spike with values up to *≈*50 mm Hg, in line with what was found in previous experiments performed in porcine eye.^20^ We note that, as further commented in the Discussion, the intensity of this pressure spike is likely to be unrealistically large in our experiments, owing to the fact that our domain is rigid.

The pressure signals obtained with the 23- and 25-gauge TDC cutters were qualitatively similar; however, the pressure drops were typically larger for the 25-gauge TDC probe, and in that case, the pressure might even reach negative values.

**Figure 5.**
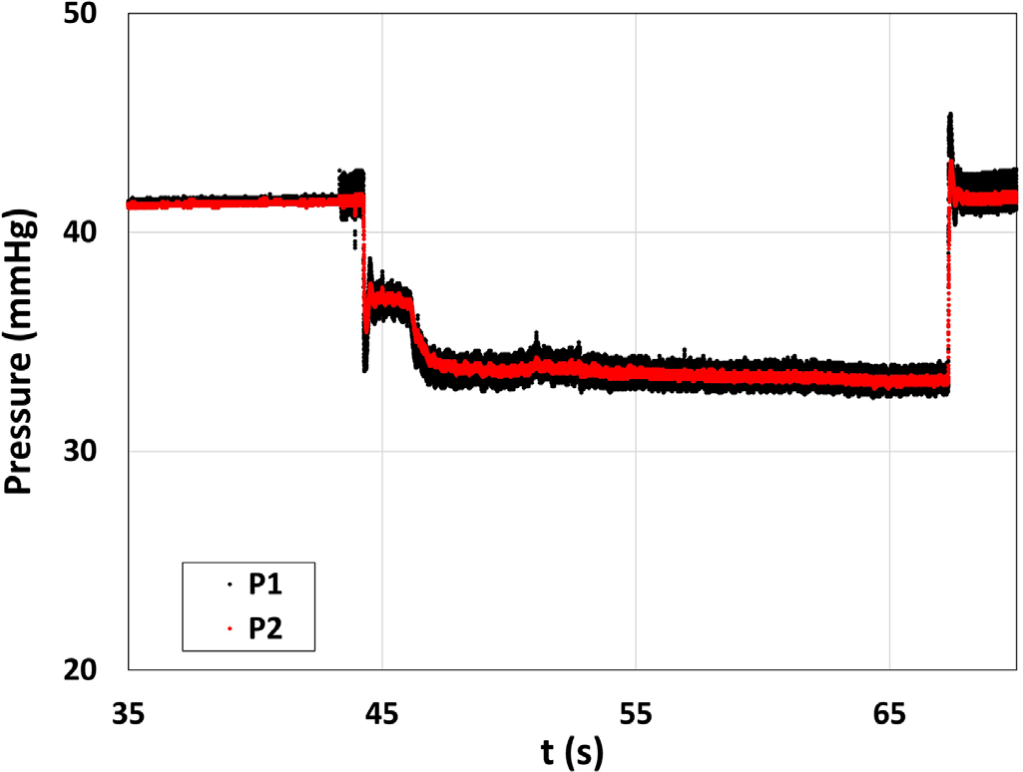
Example of pressure versus time signals, measured at two different positions of the eye model, using AV in VGPC mode. *Black curve*: posterior position (P1); *red curve*: anterior position(P2).

Figures 4b and 4d show the corresponding pressure signals in AIC mode. In this case, the system attempts to compensate the pressure drop by increasing the infusion pressure when aspiration starts. The pressure drop in AIC mode was typically reduced, and for an infusion pressure of 30 mm Hg (orange curves), compensation was very efficient. Leaving aside the differences highlighted above, the main characteristics of the experiments with AV are the same as with BSS; see Figure 5 for an example of pressure recordings during an AV experiment.

**Figure 6.**
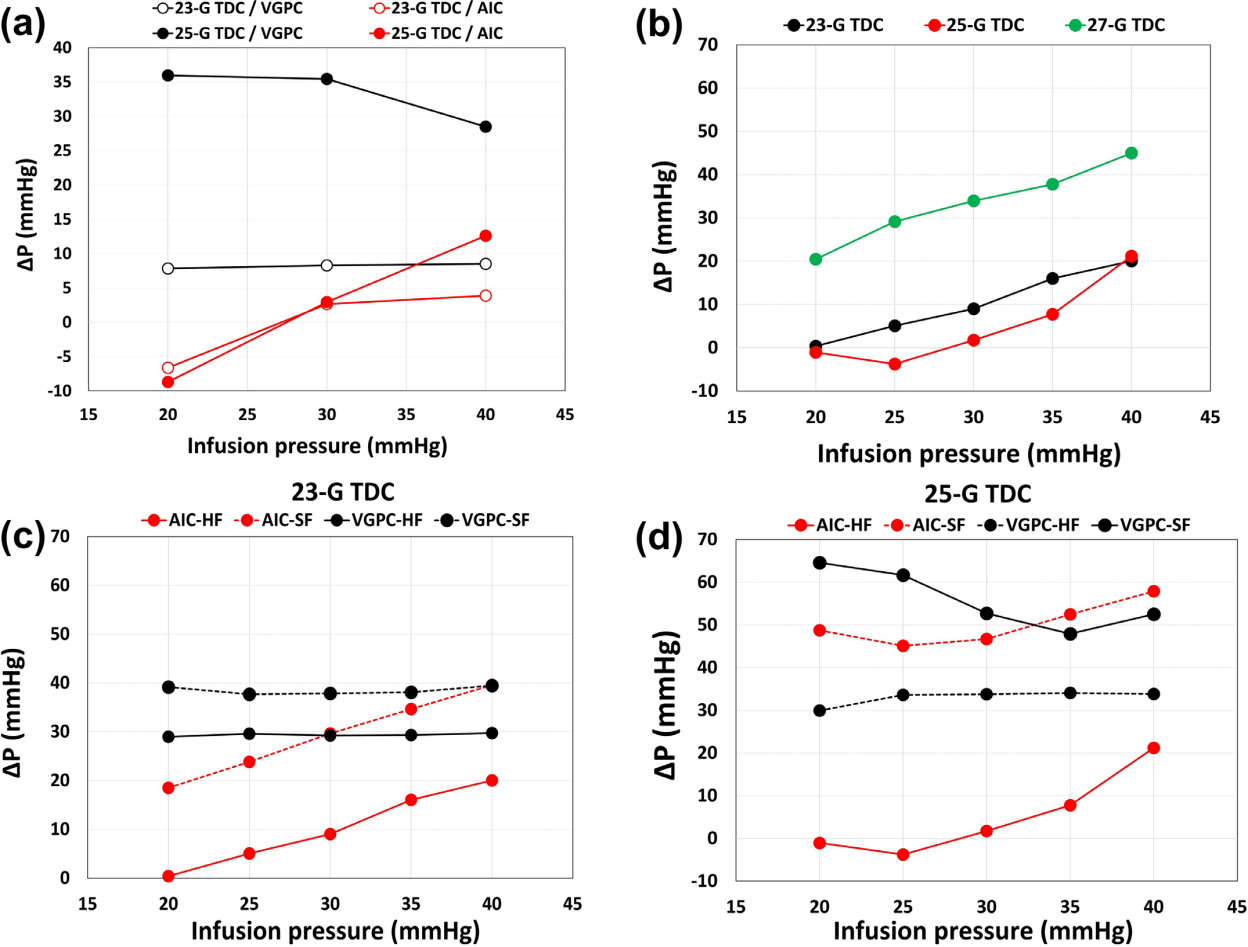
(a) Pressures drop, Δ*P*, as a function of the imposed infusion pressure. *Black curves*: VGPC mode; *red curves*: AIC mode. All experiments have been performed with AV using the HF connection. (b) Pressures drop in AIC mode, Δ*P*, as a function of the imposed infusion pressure. (c) The 23-gauge TDC cutter and (d) 25-gauge TDC cutter. All experiments shown in panels (b), (c), and d) have been performed with BSS with the HF infusion line.

A more detailed comparison between VGPC and AIC modes is reported in Figure 6a, where we show the average pressure jump (Δ*P*) versus the imposed infusion pressure. Note that Δ*P* is positive when the pressure during aspiration is smaller than the baseline value (this is always the case in VGPC mode). In VGPC mode, Δ*P* remained fairly constant with the imposed infusion pressure. In AIC mode, the pressure drop was typically smaller than in VGPC mode, especially with the 23-gauge TDC cutter. Note that pressure compensation at low infusion pressures may produce a pressure increase during aspiration (Δ*P* < 0).

For the 27-gauge TDC cutter, we performed experiments only in AIC mode, using BSS. The corresponding results are summarized and compared to those obtained with the other cutters in Figure 6b. The figure shows that compensation with the 27-gauge TDC (green curve) was less efficient than with 23- and 25-gauge TDC cutters.

**Figure 7.**
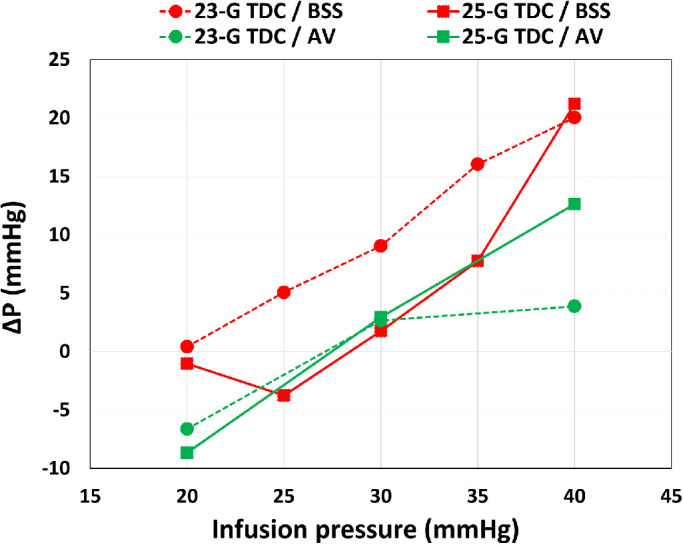
Pressures drop in AIC mode, Δ*P*, as a function of the imposed irrigation pressure. *Red curves*: experiments with BSS; *green curves*: experiments with AV. Using BSS, the 25-gauge TDC cutter achieved values very similar to those obtained with AV for the usual infusion pressures set during vitrectomy (25–30 mm Hg). In general, the 23-gauge TDC probe in BSS produced higher values of Δ*P*. For an infusion pressure set to 20 mm Hg, both cutter gauges compensated very efficiently pressure variations.

Results regarding the differences between HF and SF connections for the infusion system are summarized in Figures 6c and 6d for the 23- and 25-gauge TDC cutters, respectively. The HF connection resulted in much lower pressure jumps, Δ*P*, during aspiration. In general, pressure drop with AV was found to be smaller than with BSS with the same working parameters (see Fig. 7).

Finally, flow rate measurements demonstrated a greater aspiration efficiency when the HF connection was used for all vitreous probes and in both VGPC and AIC modes (see Table 2).

**Table 2.** Flow Rate as a Function of the Imposed Infusion Pressure for the 23- and 25-Gauge TDC Cutter

	Infusion Pressure	Flow Rate	Flow Rate	Flow Rate VGPC-HF	Flow Rate VGPC-SF
Cutter	(mm Hg)	AIC-HF (mL/min)	AIC-SF (mL/min)	(mL/min)	(mL/min)
23-gauge TDC	25	24.48	23.55	21.93	19.32
23-gauge TDC	30	23.67	23.84	21.32	19.45
23-gauge TDC	35	24.25	23.95	22.04	19.7
23-gauge TDC	40	24.42	23.91	22.74	19.72
25-gauge TDC	25	17.81	15.21	23.18	15.1
25-gauge TDC	30	17.84	15.13	21.42	13.51
25-gauge TDC	35	16.8	15.47	22.6	13.71
25-gauge TDC	40	17.04	15.39	22.15	11.79

## Discussion

The aim of the present work was to test the efficiency of the EVA phacovitrectomy system (DORC) in maintaining baseline pressure values during the various phases of the surgical procedure.

In addition, flow rate experiments have been performed with the aim of assessing the performance of EVA high-flow connection for the infusion system.

In order to evaluate the pressure drop, we computed the average pressure jump, Δ*P*, defined as Δ*P* = *P_b_* − *P_a_*, where *P_b_* and *P_a_* are the time-averaged pressures during the infusion and aspiration phases. We found that the pressure drop was reduced in AIC mode compared to VGPC mode. The baseline infusion pressure was quite efficiently maintained with both 23- and 25-gauge TDC cutters, imposing a minimum and a maximum infusion pressures of 30 and 55 mm Hg, respectively. Generally, however, the 23-gauge TDC cutter resulted in better pressure compensation during aspiration with respect to the 25-gauge TDC cutter, using AV. Pressure variations were larger using the 27-gauge TDC cutter compared to those of larger size, demonstrating that the former was less efficient in IOP compensation (tested only with BSS).

In BSS, we found on average Δ*P* values larger than those obtained with AV, especially when using the 23-gauge TDC cutters or when we imposed large values of the infusion pressure.

In some cases, compensation led to a pressure increase in the eye model (Δ*P* < 0), which is an undesirable effect. This was found, for example, when the 25-gauge TDC cutter was used in AV with a low value of the minimum infusion pressure. From a surgical point of view, it may be argued that imposing a low value of the minimum infusion pressure in the attempt to keep IOP at low levels can be a wrong operating strategy. This is particularly relevant in eyes where high intraoperative IOP and the consequent ocular hypoperfusion may be harmful, such as cases in which blood flow is already compromised (e.g., diabetic retinopathy, retinal vein occlusion, or advanced glaucoma).^20^^,^^44^^,^^45^ This effect could be avoided by setting a lower value of the maximum infusion pressure in AIC mode. The ideal values range for the infusion pressure in AIC depends on the rheological properties of the vitreous humor and the aspiration pressure.

A pressure spike was always found when aspiration ceased at the end of phase 2. This behavior was likely enhanced by the fact that we operated in a rigid domain, and the only compliance was due to the pipes. Thus, such an effect is expected to be reduced in real eyes, which are compliant. We also note that Sugiura et al.^20^ suggested that relatively large IOP spikes up to 10 to 20 mm Hg may not have clinical relevance when their duration is very limited. Despite the rigid material of the model, the present pressure measurements are in line with previous studies performed on porcine eye.^21^^,^^23^^,^^24^ We also note that our main interest is in pressure values attained in the eye model during “steady” phases of the experiment, during which the aspiration pressure is maintained fixed over time. In this case, the pressure in the eye model is not expected to be significantly affected by the model material properties.

During vitrectomy, the cutter produces high-frequency pressure fluctuations that can be harmful.^11^ In this study, dynamic variations of the pressure due to the motion of the cutter blade have been measured and always found to be small compared with the average pressure in the eye model. However, inspection of the pressure signals shows that the amplitude of pressure fluctuations is larger at the back pole than in the front region of the vitreous chamber model. This observation means that the posterior pressure probe is more affected by the flow than the anterior one, in agreement with previous studies.^16^ Translated to the surgical practice, this last finding suggests that surgeons should be aware that pressure fluctuations due to the jet of the infusion line could lead to a focal chorioretinal damage.^46^

Finally, we also carried out flow rate experiments in BSS to compare the performance of HF and SF connections for the infusion line. The purpose of the HF configuration is to maximize the flow rate, as the infusion line is connected to the external part of the trocar-cannula, thus exploiting the entire section of the pipe. Consistently with the intended use, the HF infusion system was found to be more efficient in terms of flow rate, with both 23- and 25-gauge TDC probes. In particular, using the 25-gauge TDC cutter, we obtained an increase in flow rate up to *≈*20% and *≈*70% higher than the SF configuration, in AIC and VGPC mode, respectively. Moreover, the flow rates achieved with the HF connection were significantly larger compared to those found in previous studies with a different vitrectomy device.^24^ From the surgical point of view, this finding is important since within a closed system, such as the eye during vitrectomy, the use of instruments of progressively smaller diameter can result in the need to increase the infusion pressure in order to maintain a satisfactory flow rate.^47^ However, a sustained increase of infusion pressure and intraoperative IOP is not advisable due to the negative effect on the mean ocular perfusion pressure and, thus, potential ischemic damage.^14^ Therefore, HF can optimize vitrectomy fluidics, allowing the operator to obtain large flows and keeping the infusion pressure within a safe range, also with smaller gauge instruments. When the HF configuration was used, smaller values of Δ*P* were found with respect to the SF connection for the same operating conditions, meaning that this infusion system also improves pressure stability during surgery.

## Conclusions

In the present study, we measured dynamic intraocular pressure variations during vitrectomy in a real scale model of the vitreous chamber, using both balance salt solution and artificial vitreous, in order to simulate a realistic surgical scenario in a controlled environment. We demonstrated that the AIC mode of the EVA phacovitrectomy system was quite effective in compensating the pressure drop (∆*P*) generated by the aspiration and in ensuring more stable IOP values close to the imposed infusion pressure, especially when the infusion pressure was set at 30 mm Hg, for both 23- and 25-gauge TDC cutters. However, the present results showed that in some cases, typically at low infusion pressures, compensation may lead to excessive pressure values in the eye model. IOP compensation was found to be less efficient with the 27-gauge TDC cutter.

The HF infusion system performed better than the SF infusion system, resulting in higher flow rate and lower pressure fluctuations, Δ*P*. Therefore, the combined use of HF and AIC can optimize the inflow and the predictability of real intraoperative IOP. With regard to surgical practice, this study contributes to better define operating conditions that improve intraoperative stability of the vitreous chamber. We acknowledge that using a rigid vitreous chamber model is a limitation of the present study. However, we consider this an essential step toward more realistic models that consider the complaint characteristic of the human eye.
